# Oxidative stress regulates cellular bioenergetics in esophageal squamous cell carcinoma cell

**DOI:** 10.1042/BSR20171006

**Published:** 2017-12-12

**Authors:** Xiaolong Zhang, Linhua Lan, Lili Niu, Juping Lu, Changxi Li, Miaomiao Guo, Shouyong Mo, Jing Lu, Yongzhang Liu, Bin Lu

**Affiliations:** 1 Department of Anesthesia and Critical Care, The Second Affiliated Hospital and Yuying Children’s Hospital of Wenzhou Medical University, Wenzhou, Zhejiang 325027, China; 2 Protein Quality Control and Diseases Laboratory, Cancer Center, School of Laboratory Medicine and Life Sciences, Wenzhou Medical University, Wenzhou, Zhejiang 325035, China; 3 Department of Biochemistry, Institute of Biophysics, Attardi Institute of Mitochondrial Biomedicine and Zhejiang Provincial Key Laboratory of Medical Genetics, School of Life Sciences, Wenzhou Medical University, Wenzhou, Zhejiang 325035, China; 4 Department of Laboratory Medicine, The Fifth Affiliated Hospital of Wenzhou Medical University, Lishui, Zhejiang 32300, China

**Keywords:** bioenergetics, esophageal squamous cell carcinoma, hypoxia, mitochondria, oxidative stress, reactive oxygen species

## Abstract

The aim of the present study was to explore the effects of oxidative stress induced by CoCl_2_ and H_2_O_2_ on the regulation of bioenergetics of esophageal squamous cell carcinoma (ESCC) cell line TE-1 and analyze its underlying mechanism. Western blot results showed that CoCl_2_ and H_2_O_2_ treatment of TE-1 cells led to significant reduction in mitochondrial respiratory chain complex subunits expression and increasing intracellular reactive oxygen species (ROS) production. We further found that TE-1 cells treated with CoCl_2_, a hypoxia-mimicking reagent, dramatically reduced the oxygen consumption rate (OCR) and increased the extracellular acidification rate (ECAR). However, H_2_O_2_ treatment decreased both the mitochondrial respiration and aerobic glycolysis significantly. Moreover, we found that H_2_O_2_ induces apoptosis in TE-1 cells through the activation of PARP, Caspase 3, and Caspase 9. Therefore, our findings indicate that CoCl_2_ and H_2_O_2_ could cause mitochondrial dysfunction by up-regulation of ROS and regulating the cellular bioenergy metabolism, thus affecting the survival of tumor cells.

## Introduction

Esophageal squamous cell carcinoma (ESCC) is the most frequent histological subtype of esophageal cancer and is the sixth most common cause of cancer-associated mortality worldwide [1]. Despite advances in early diagnosis, surgery, and chemoradiotherapy, the prognosis of ESCC is still very poor and remains a challenge [2]. Thus, our understanding of mechanisms of ESCC tumorigenesis is urgent, which will facilitate the development of diagnosis marker and novel therapeutic strategies.

Unlike normal cells, tumor cells demand a vast amount of energy (ATP) and metabolites to support their rapid proliferation. Indeed, some tumor cells are addicted to glycolysis rather than oxidative phosphorylation (OXPHOS) even in normoxic conditions and need quick reprogramming of energy metabolism. This phenomenon is often referred to as the Warburg effect [3]. Importantly, glycolysis provides cancer cells with not only energy but also crucial intermediates for biosynthesis of macromolecules, lipids as well as NADPH which is important for redox homeostasis. Metabolic reprogramming is required for cell malignant transformation, tumorigenesis, invasion, metastasis, and resistance to cancer treatments [4]. In solid tumor, hypoxia is a crucial microenvironmental stimuli that leads to significant up-regulation of hypoxia-inducible factor-1α (HIF-1α) in majority of human cancers [5–7]. Cobalt chloride (CoCl_2_), a well-known hypoxia-mimicking agent *in vitro*, which blocks HIF-1α degradation and thus causes HIF-1α accumulation in cells, therefore leading to an intracellular hypoxia-like microenvironment and enhancing tumor malignancy.

Because hydrogen peroxide (H_2_O_2_) can generate large amounts of oxygen free radicals and cause oxidative stress in tumor cells, it is widely used as an apoptosis inducer [8]. Indeed, many anticancer drugs promote cancer cell death through enhancing the intracellualr H2O2 generation [9,10].Thus, studying the effects of H_2_O_2_ on cancer cells is helpful to provide novel strategies for the prevention and treatment of cancers.

H_2_O_2_ is also an important signaling molecule in tumor cells, which can regulate cell signaling pathways and transcription factors at different levels [11,12]. For example, H_2_O_2_ can induce the activation of EGFR, which leads to the amplification of Ras signaling cascade and activation of mitogen-activated protein kinases (MAPKs) [13]. Moreover, H_2_O_2_ can indirectly regulate PTK-EGFR-Ras signaling in cancer cells, and finally the activation of MAPK, so that ERK, JNK, p38, three subfamilies signaling pathways are regulated [13,14].

Low concentration of H_2_O_2_ plays an essential role in regulating cell division and growth as a signaling molecule [15,16]; however, when the amount of H_2_O_2_ exceeds a certain critical value, the cell cycle will be blocked and even lead to apoptosis [17]. Therefore, the up-regulation of H_2_O_2_ amount in tumor cells is one of the strategies to kill tumor cells. This process can also be rescued by up-regulation of catalase expression. Overexpression of SOD inhibits tumor cell proliferation, metastasis, and other malignant phenotypes. Similarly, catalase or glutathione reductase can reverse the antitumor effect of SOD [18].

In the present study, we examined the effects of CoCl_2_-induced hypoxia and H_2_O_2_ on the regulation of cellular bioenergy metabolism in ESCC cells and sought to investigate the underlying molecular mechanism.

## Materials and methods

### Reagents and antibodies

RPMI-1640 medium and FBS (Life Technologies, Grand Island, NY); oligomycin, FCCP, rotenone, antimycin A, glucose, 2-DG, and CoCl_2_ were purchased from Sigma (St. Louis, MO); Annexin V-FITC/propidium iodine (PI) apoptosis detection kit was from BD Pharmingen (San Diego, CA); 2′,7′-dichlorodihydrofluorescein diacetate (DCFH-DA), horseradish peroxidase (HRP) conjugated anti-rabbit, anti-mouse Ig, trypsin were from Beyotime (Shanghai, China). The following antibodies were used: anti-COXI, anti-COXII, anti-COXIV, and anti-SDHA (Abcam, Cambridge, MA); anti-ND2, anti-NDUFA5, anti-NDUFA6, anti-NDUFA9, anti-HIF-1α, anti-ATP5A, anti-PARP, anti-Caspase-9, anti-Caspase-3, anti-cleaved Caspase-3 (Cell Signaling, Beverly, MA); anti-β-actin (Abmart, Shanghai, China); anti-Cyt. b (Santa Cruz Biotechnology, Santa Cruz, CA). All other chemicals used were of highest analytical grade and obtained from Sigma, unless otherwise stated.

### Cell lines and culture conditions

The human ESCC cell line TE-1 was obtained from the Cell Bank of the Chinese Academy of Sciences (Shanghai, China). TE-1 cells were cultured in RPMI-1640 medium supplemented with 10% FBS and antibiotics (100 units/ml penicillin and 100 μg/ml streptomycin) at 37°C with 5% CO_2_.

### Determination of alterations in cell morphology and cell number

TE-1 cells were plated in 60-mm dishes at a density of 0.3 × 10^6^ and incubated with series concentrations of H_2_O_2_ for 24 h at 37°C with 5% CO_2_. Inverted microscopy (Nikon, Tokyo, Japan) was used to examine the alterations in cell morphology and cell number.

### FACS analysis for intracellular ROS

Cells were harvested by trypsin, washed with PBS, and incubated with DCFH-DA at a final concentration of 10 µM in RPMI-1640 medium for 30 min at 37°C, then cells were washed three times with cold PBS. Intracellular reactive oxygen species (ROS) levels were measured as described previously by using the fluorescence probe DCFH-DA according to the manufacturer’s protocol (Beyotime, Shanghai, China) [19]. The excitation wavelength for DCF fluorescence is 488 nm and the emission wavelength for DCF fluorescence is 525 nm, respectively.

### FACS analysis for apoptosis

Harvested cells were washed with cold PBS twice, and incubated with 1× binding buffer at a concentration of 1 × 10^6^ cells/ml. Annexin V-FITC/PI (BD, San Jose, CA) was added, followed by incubation in the dark at room temperature for 20 min. Four hundred microliters of 1X binding buffer was added to each tube. The samples were analyzed by flow cytometry within 1 h.

### Immunoblotting

Cells were washed with cold PBS and lysed in RIPA lysis buffer supplemented with protease and phosphatase inhibitors on ice for 20 min, followed by centrifugation at 13000 rpm for 20 min at 4°C, and the supernatants were collected. Protein concentrations of whole cell extracts were determined using the Pierce BCA protein assay kit. Cell extracts equivalent to 20 µg total protein were resolved in 10% SDS/PAGE gels followed by electrophoretic transfer on to nitrocellulose membrane (Bio–Rad, Hercules, CA) in Tris-glycine buffer. Blots were blocked at room temperature for 2 h in 3% non-fat milk in TBS-Tween (TBS-T) on a shaker, and then incubated with the primary antibodies overnight at 4°C. The membrane was washed in TBS-T for at least 3× forw 10 min and then incubated with HRP conjugated anti-rabbit or anti-mouse IgG at room temperature for 1 h with gentle shaking. Signal was detected using ECL according to the manufacturer’s protocol (Thermo Scientific, Rockford, IL) and exposed to X-ray films. β-actin was used as control for equal loading and the optical density was measured using National Institute of Health ImageJ software. Each experiment was repeated at least three times.

### RNA preparation and quantitative real-time PCR analysis

Total RNAs were extracted from cells using TRIzol reagent (Life Technologies, Carlsbad, CA) following manufacturer’s instructions. cDNA was synthesized from 2 μg purified total RNA using the PrimeScript™ RT reagent Kit with gDNA Eraser (Takara, Dalian, China). Quantitative real-time PCR (qRT-PCR) was performed as described previously [19] using gene-specific primers. The results were normalized to β-actin in the same samples. Each sample was analyzed in triplicate and repeated three times. The primer sequences used were as follows: *COXI* 5′-TCCGCTACCATAATCATCGCT-3′ (forward), 5′-CCGTGGAGTGTGGCGAGT-3′ (reverse); *COXII* 5′-CGACTACGGCGGACTAATCT-3′ (forward), 5′-TCGATTGTCAACGTCAAGGA-3′ (reverse); *COXIV* 5′-GCAGTGGCGGCAGAATG-3′ (forward), 5′-AGTCTTCGCTCTTCACAACA-3′ (reverse); *ATPase6* 5′-*CTGTTCGCTTCATTCATTGC*-3′ (forward), 5′-AGTCATTGTTGGGTGGTGAT T-3′ (reverse), ATPase8 5′-AAACTACCACCTACCTCCCTCAC-3′ (forward), 5′-GCAATGAATGAAGC ATTCATTGC-3′ (reverse), ND3 5′-GCGGCTTCGACCCTATATC-3′ (forward), 5′-TGGCAGGTTAGTT GTTTGTAGG-3′ (reverse), ND4 5′-TGAACGCAGGCACATACTTC-3′ (forward), 5′-TCTTGGGCAGT GAGAGTGAG-3′ (reverse), ND5 5′-CATTGTCGCATCCACCTTTA-3′ (forward), 5′-CTGGGTTGTTTGG GTTGTG-3′ (reverse), ND6 5′-GGTGCTGTGGGTGAAAGAGT-3′ (forward), 5′-CTCCCGAATCAA CCCTGAC-3′ (reverse), β-actin 5′-GCCAGTGGACTCCACGAC-3′ (forward), 5′-CAACTACATGGTTTA CATGTTC-3′ (reverse).

### OXPHOS and glycolysis assay

The intact cellular oxygen consumption rate (OCR) and extracellular acidification rate (ECAR) of TE-1 cells were measured using a Seahorse XF-96 Extracellular Flux Analyzer (Seahorse Bioscience, North Billerica, MA) as previously described [19]. Results were obtained by performing three independent experiments with eight replicates. After the assay was completed, a BCA Protein Assay Kit was used according to the manufacturer’s instructions to determine the protein concentration to normalize OCR and ECAR.

### Statistical analysis

All experiments were performed at least three times and the data obtained were analyzed using SPSS version 16.0 (SPSS, Chicago, U.S.A.). The values are presented as the mean ± S.D. For the evaluation of two groups, Student’s *t* test was used. *P*<0.05 was considered to indicate a statistically significant difference.

## Results

### Effects of CoCl_2_ on the expression of mitochondrial respiratory chain complex subunits

**Figure 1:**
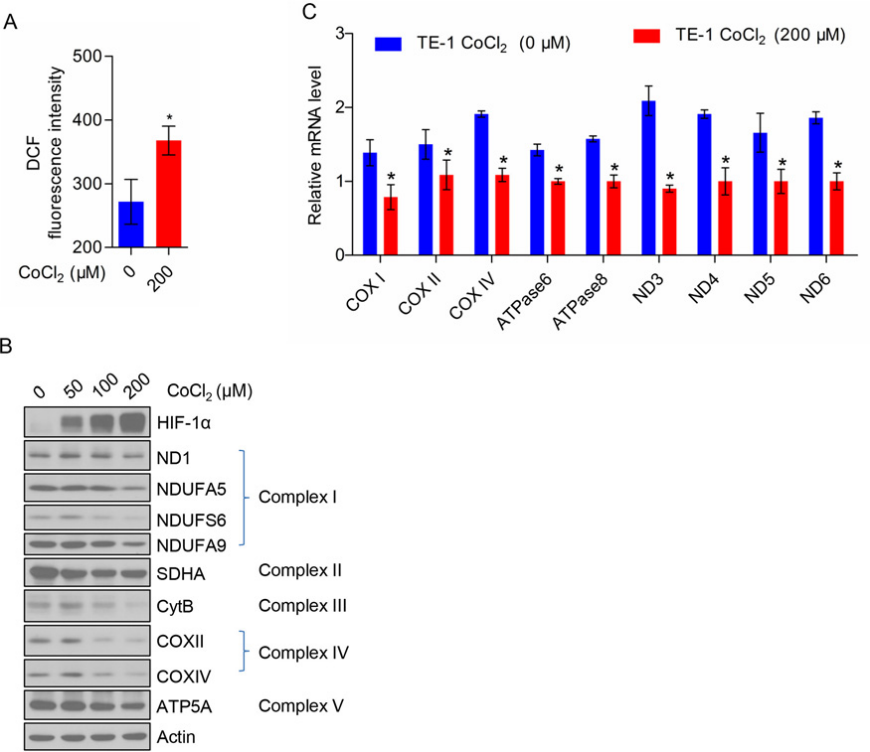
CoCl_2_ inhibits the expression of mitochondrial respiratory chain complex subunits (**A**) CoCl_2_ (200 μM) induces ROS production in TE-1 cells. (**B**) The expression profile of mitochondrial respiratory chain complex subunits and HIF-1α in TE-1 cells treated with a gradient concentration of CoCl_2_. (**C**) CoCl_2_ (200 μM) reduces the mRNA level of mitochondrial respiratory chain complex subunits of TE-1 cells.

To determine the effect of CoCl_2_ on TE-1 cells, we first measured the intracellular ROS level in CoCl_2_-treated TE-1 cells by flow cytometry. CoCl_2_ treatment significantly increased the ROS production of TE-1 cells as shown in Figure 1A. Next, we treated TE-1 cells with the indicated concentration of CoCl_2_ (0, 50, 100, and 200 μM) and analyzed the expression of HIF-1α, complex I subunits (ND1, NDUFA5, NDUFS6, and NDUFA9), complex II subunit (SDHA), complex III subunit (Cyt. B), complex IV subunits (COX II and COX IV) and complex V subunit (ATP5A) in TE-1 cells by Western blot. We found that the expression of HIF1-α protein increased in a dose-dependent manner of COCl_2_. However, the expression of ND1, cytochrome *b* and COX II encoded by mtDNA and NDUFA5, NDUFS6, NDUFA9, SDHA, COX IV, and ATP5A encoded by nuclear gene were decreased in a dose-dependent manner of CoCl_2_ treatment (Figure 1B). To clarify whether CoCl_2_ regulates the protein expression or transcription, we further examined the mRNA levels of these proteins (Figure 1C).

Taken together, our findings indicated that CoCl_2_ may inhibit mitochondrial respiration in TE-1 cells.

### Effect of CoCl_2_ on TE-1 cell bioenergetics metabolism

**Figure 2:**
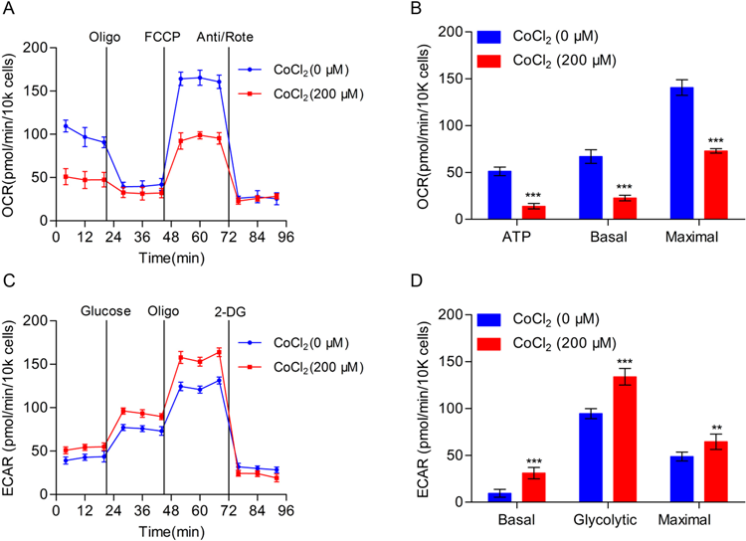
The effect of CoCl_2_ on bioenergetics metabolism in TE-1 cells (**A**) TE-1 cells with or without CoCl_2_ (200 μM) treatment for 24 h, and the OCR was measured real-time using Seahorse XF96 Extracellular Flux analyzer. The basal OCR was measured at three time points, and then four chemicals were injected into the medium sequentially: the ATP synthase inhibitor oligomycin (1 μM), the uncoupler FCCP (1 μM), the complex I inhibitor rotenone (1 μM), and complex III inhibitor antimycin (1 μM). (**B**) Statistical analysis of OCR in TE-1 cells with or without CoCl_2_ (200 μM) treatment. ATP production, basal, and maximal respiration were presented as mean ± S.D. of six replicates. (**C**) TE-1 cells treated with or without CoCl_2_ (200 μM) treatment for 24 h. ECAR was detected by the Seahorse XF96 Extracellular Flux Analyzer. Three drugs were added sequentially: glucose (10 mM), oligomycin (1 μM), and 2-DG (100 mM). (**D**) Statistical analysis of ECAR in TE-1 cell with or without CoCl_2_ (200 μM) treatment. Basal ECAR, glycolytic ECAR, and maximal ECAR are presented as mean ± S.D. of six replicates; **P<0.01, ***P<0.001.

In order to further study the effect of CoCl_2_ on cellular bioenergetics metabolism, we used Seahorse XF96 Extracellular Flux Analyzers to detect the OCR and found that OCR in TE-1 cells decreased significantly after treating with CoCl_2_ for 24 h (Figure 2A). The production of ATP, basal respiration, and maximal respiration was markedly reduced and the difference was statistically significant (Figure 2B). In addition, we detected the ability of glycolysis in TE-1 cells when treated with CoCl_2_, as result showed that when compared with the negative control, the glycolysis ability of TE-1 cells significantly increased under the treatment of CoCl_2_ and the difference was statistically significant (Figure 2C,D).

### NAC could rescue the effect of CoCl_2_ on the expression of mitochondrial respiratory chain complex subunits and bioenergetics metabolism of TE-1 cells

**Figure 3:**
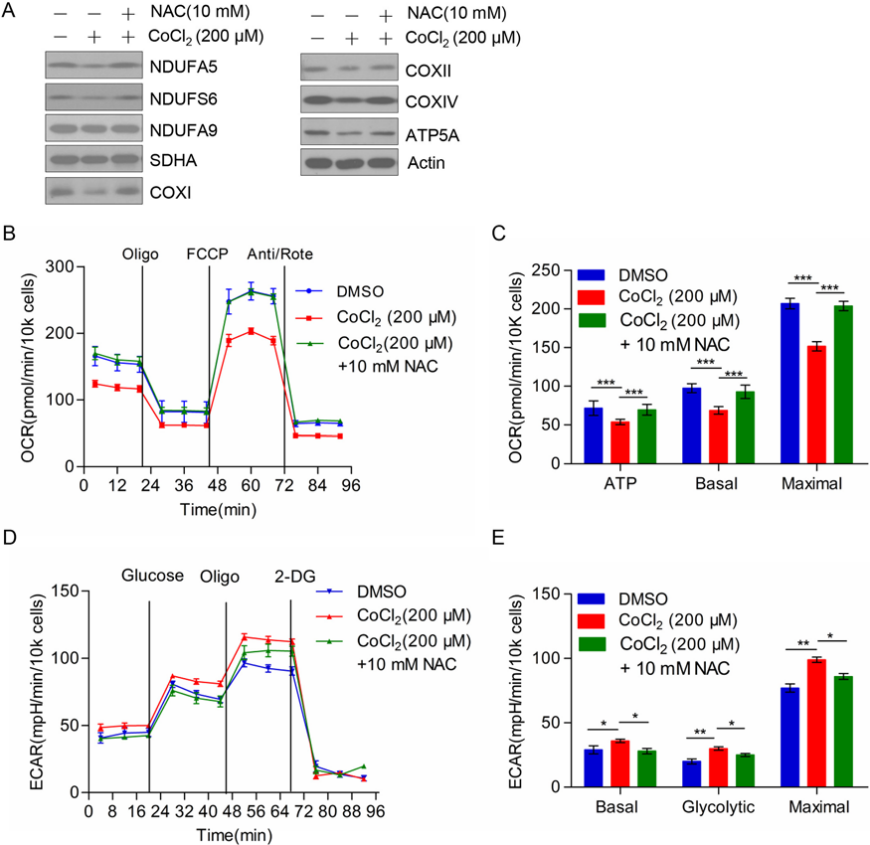
NAC rescued the effect of CoCl_2_ on the expression of mitochondrial respiratory chain complex subunits and bioenergetics metabolism in TE-1 cells (**A**) The expression of HIF-1α and mitochondrial complex subunits in control group (DMSO), CoCl_2_ (200 μM) treated group, CoCl_2_ (200 μM), and NAC (10 mM) treated group. (**B**) OCR of control group (DMSO), CoCl_2_ (200 μM) treated group, CoCl_2_ (200 μM), and NAC (10 mM) treated group in TE-1 cells. (**C**) ATP production, basal respiration, and maximal respiration were presented as mean ± S.D. of six replicates. Quantitation and statistical analysis of data from (B). (**D**) Overall ECAR of TE-1 cells which was treated by CoCl_2_ in the presence or absence of NAC. (**E**) Basal glycolytic rate, spare glycolytic, and maximal glycolytic rate were assessed according to the manufacturer’s protocol; *P<0.05, **P<0.01, ***P<0.001.

HIF-1α was one of the important transcription factors in tumor development and progression, contributed to cell survival, and activation of gene expression under hypoxic condition. The target genes mainly related to metabolism of carbohydrates that include glycolytic enzymes, aldolase A, and glucose transporter protein-1 (GLUT-1). We hypothesized that ESCC cell TE-1 may switch cellular energy metabolism from mitochondrial OXPHOS to glycolysis under hypoxic conditions stimulated by CoCl_2_. On the one hand, TE-1 cells inhibited the expression of mitochondrial complex subunits by increasing ROS level; on the other hand, TE-1 cell enhanced glycolysis ability by increasing the expression of glucose metabolism related enzymes. To demonstrate our hypothesis, we set three groups: the negative control group, CoCl_2_ treated group, and both CoCl_2_ and N-acetyl cysteine (NAC, ROS scavenger) treated group. Western blot was used to detect mitochondrial complex subunits protein expression in the three groups. We found that the subunits of mitochondrial complex recovered obviously in the group of TE-1 cells treated with CoCl_2_ and NAC simultaneously (Figure 3A). Meanwhile, by using Seahorse Bioenergetics Analyzer to measure OCR, we found that NAC could significantly rescue mitochondrial respiration in TE-1 cells treated with CoCl_2_ (Figure 3B). The difference was statistically significant (Figure 3C). Additionally, we found that NAC could rescue CoCl_2_ induced up-regulation of aerobic glycolysis. As shown in Figure 3D, NAC treatment decreased aerobic glycolysis enhanced by CoCl_2_. Moreover, we assessed the basal glycolytic rate, spare glycolytic, and maximal glycolytic rate, which indicated that NAC treatment could reverse CoCl_2_ induced aerobic glycolysis (Figure 3E). Hence these results suggested that ESCC cell TE-1 maintained cell survival by the transformation of energy metabolism under conditions of low oxygen caused by CoCl_2_.

### H_2_O_2_ induced TE-1 cells death through generation of ROS

**Figure 4:**
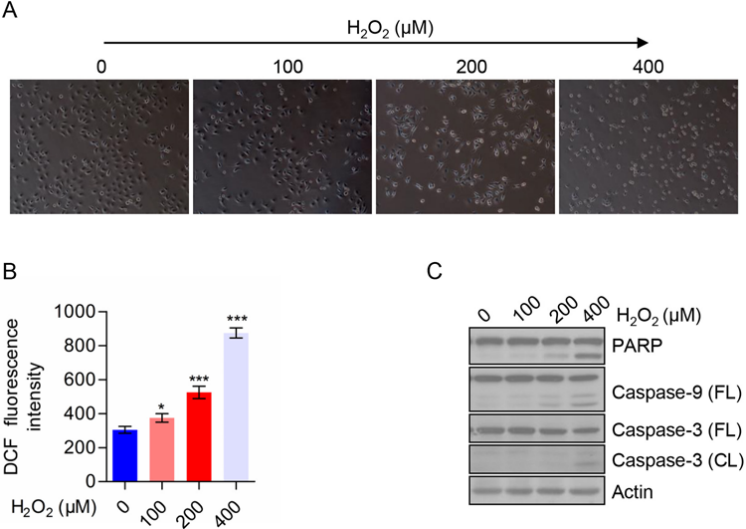
H_2_O_2_ induced TE-1 cells death through generation of ROS (**A**) Morphology of TE-1 cells treated with the indicated concentrations of H_2_O_2_. (**B**) ROS level of TE-1 cells treated with the indicated concentration of H_2_O_2_. (**C**) The expression of apoptosis-related proteins in TE-1 cells treated with the indicated concentrations of H_2_O_2_. All data are presented as mean ± S.D.; *P<0.05, ***P<0.001.

As shown in Figure 4A, we treated TE-1 cells with the indicated concentration of H_2_O_2_. We observed cell morphological changes under phase contrast microscopy. With the increase in the concentration of H_2_O_2_, the cell viability was decreased compared with the control group as well as the cell size became smaller, indicating that the cells tended to apoptotic state. To explore the effect of H_2_O_2_ on TE-1 cells, we analyzed the intracellular ROS level by flow cytometry. The result showed that compared with the negative control group, intracellular ROS level increased in a dose-dependent manner in H_2_O_2_-treated TE-1 cells (Figure 4B). In order to further examine the apoptotic rate of cells, we detected the apoptosis-related proteins by Western blot and the results showed that cleaved-PARP, cleaved-Caspase 3, and cleaved-Caspase 9 were increased in a dose-dependent manner with H_2_O_2_ treatment from 100 to 400 μM (Figure 4C). These data indicated that H_2_O_2_ could induce TE-1 cells death that is mediated through caspase activation. We also analyzed the effects of H_2_O_2_ and CoCl_2_ on cell growth, and found that both H_2_O_2_ and CoCl_2_ could suppress cell growth in TE-1 cells. However, H_2_O_2_ had a stronger suppressive activity than CoCl_2_ on TE-1 cells (Supplementary Figure S1). We further detected the cell death of CoCl_2_-treated cells, however, the treatment of CoCl_2_ had no significant effect on cell death of TE-1 cells (Supplementary Figure S2).

### H_2_O_2_ reduced the expression of mitochondrial respiratory chain complex subunits and bioenergetics metabolism in TE-1 cells

**Figure 5:**
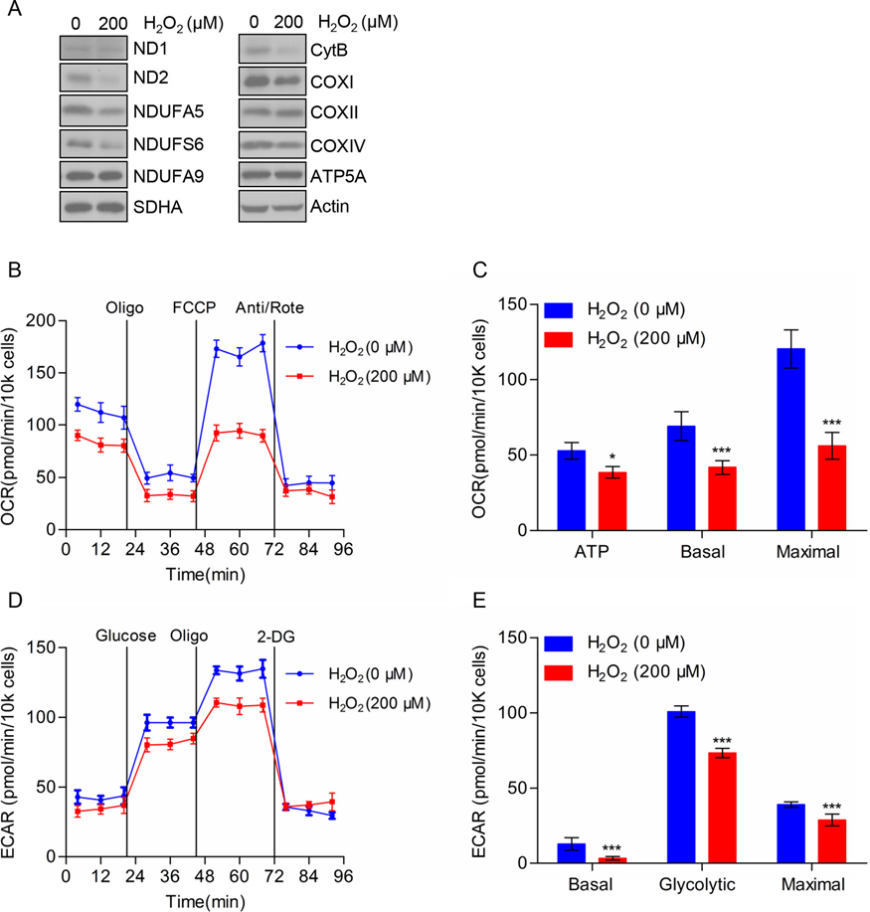
Effects of H_2_O_2_ on the expression of mitochondrial respiratory chain complex subunits and bioenergetics metabolism of TE-1 cells (**A**) TE-1 cells were pretreated with or without 200 μM H_2_O_2_ for 24 h and the cells were subjected to Western blot analysis. (**B**) OCR of TE-1 cells treated with H_2_O_2_ (200 μM). (**C**) ATP production, basal, and maximal respiration are presented as mean ± S.D. of six replicates. (**D**) ECAR of TE-1 cells treated with H_2_O_2_ (200 μM). (**E**) Quantitation of the ECAR performed on TE-1 cells as shown in (B). The data are presented as mean ± S.D.; *P<0.05, ***P<0.001.

**Figure 6:**
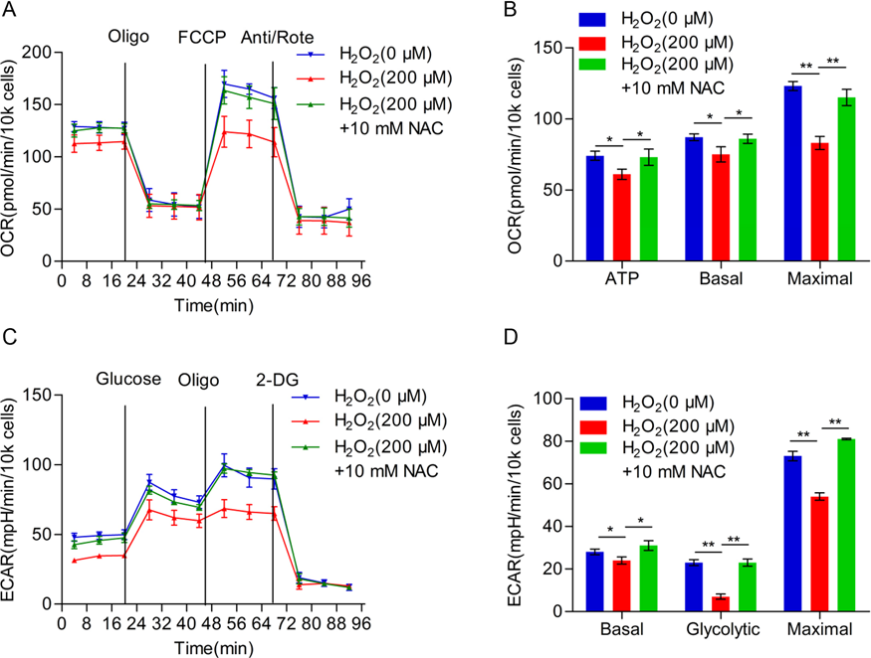
NAC rescues H_2_O_2_ caused reduction in cellular bioenergetics in TE-1 cells (**A**) Overall OCR curve of TE-1 cells treated with H_2_O_2_ (200 μM) or H_2_O_2_ (200 μM) combined with NAC (10 mM). (**B**) ATP production, basal respiration, and maximal respiration alterations of TE-1 cells in (A). (**C**) Overall ECAR curve of TE-1 cells treated with H_2_O_2_ (200 μM) or H_2_O_2_ (200 μM) combined with NAC (10 mM). (**D**) Basal glycolytic rate, spare glycolytic rate, and maximal glycolytic rate were assessed according to the treatment as shown in (C); *P<0.05, **P<0.01.

Next, we assessed the protein level in mitochondrial respiratory chain complex subunits in TE-1 cells treated with H_2_O_2_ by using Western blot analysis. As shown in Figure 5A, the expression of complex І subunits (ND2, NDUFA5, NDUFS6, and NDUFA9), complex III subunit Cyt. B and complex IV subunits (COXI and COXIV) were decreased in TE-1 cells treated with H_2_O_2_. However, there were no significant difference in the protein expression level of complex II subunit SDHA and complex V subunit ATP5A. To further investigate the effects of H_2_O_2_ on TE-1 cellular bioenergetics, we analyzed mitochondrial respiration by determining the OCR (Figure 5B), and found that cells under the treatment of H_2_O_2_ displayed a lower basal respiration and a significantly lower maximum respiratory capacity and accompanied by less ATP production compared with the negative control group (Figure 5C). In addition, we found that the glycolysis rates of TE-1 cells were significantly decreased after H_2_O_2_ treatment (Figure 5D,E). To further confirm the reduction in respiration and aerobic glycolysis were caused by H_2_O_2_ treatment, we used ROS scavenger NAC to investigate whether NAC could rescue these effects. As shown in Figure 6A, we found that NAC treatment dramatically reverses H_2_O_2_ induced mitochondrial respiration reduction, as well as ATP production, basal respiration, and maximal respiration (Figure 6B). Moreover, we detected the alterations in ECAR, and found that NAC could also rescue aerobic glycolysis suppressed by H_2_O_2_ (Figure 6C). In addition, basal glycolytic rate, spare glycolytic rate, and maximal glycolytic rate were assessed (Figure 6D). Consistently, NAC could reverse these three indexes of aerobic glycolysis. These results indicated that H_2_O_2_ could cause the reduction in cellular bioenergetics of TE-1 cells via decreasing the expression of mitochondrial respiratory chain complex subunits.

### H_2_O_2_ promotes apoptosis which could be blocked by NAC

**Figure 7:**
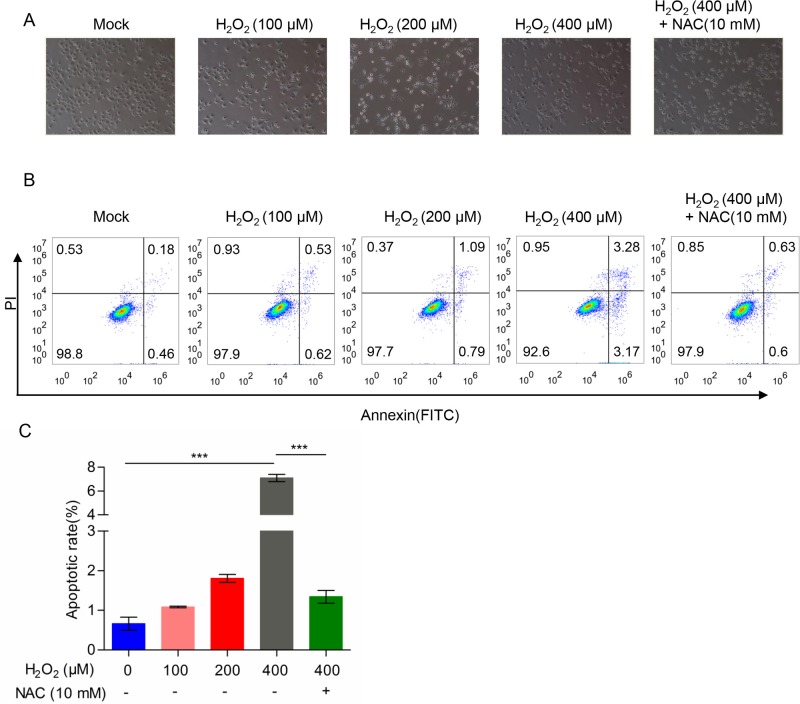
H_2_O_2_ induced the apoptosis and NAC could dramatically rescue H_2_O_2_-induced apoptosis in TE-1 cells (**A**) Morphology of TE-1 cells treated with H_2_O_2_ in the absence or presence of NAC. (**B**) TE-1 cells were pretreated with a gradient concentration of H_2_O_2_ (0, 100, 200, 400 μM) or 400 μM H_2_O_2_ and 10 mM NAC. (**C**) Cell apoptotic rate determined by flow cytometry; ***P<0.001.

Based on the above data, we speculated that H_2_O_2_ induced apoptosis in TE-1 cells may be due to cellular energy metabolism disorders that were caused by the increase in ROS. Therefore, we tested the hypothesis by adding ROS scavenger (NAC) simultaneously, and as shown in Figure 7A, TE-1 cells were treated with H_2_O_2_ in the absence or presence of NAC, and we observed that apoptosis reduced obviously in the presence of NAC. Moreover, we examined the apoptosis by flow cytometry, with the increased concentration of H_2_O_2_, the apoptosis rate gradually increased, but when TE-1 cells were treated with NAC simultaneously, we found that apoptosis induced by H_2_O_2_ was significantly reduced (Figure 7B,C). Thus, we believed that H_2_O_2_ may induce the production of ROS and cause progressive oxidative damaged, mitochondrial dysfunction, cell bioenergetics metabolism disorders, and ultimately cell death.

## Discussion

Accumulating studies have shown that the development of malignant tumors is actually a process of tumor cell metabolism reprogramming [20,21], which is one of the hallmarks of most human tumors [22]. Mitochondria as key actors in cancer metabolic reprogramming, not just because these organelles play a significant role in energy production [23] and biosynthetic intermediates formation, as well as occurrence of mutations in both nuclear and mtDNA encoding metabolic enzymes is associated with different types of cancer [24,25]. Glycolysis is an oxygen-independent metabolic pathway that converts glucose into pyruvate via a series of intermediate metabolites and leads to a net production of ATP [26]. OXPHOS is the metabolic pathway in which cells use enzymes to oxidize nutrients, thereby releasing energy which is accounts for high ATP yield [27]. OXPHOS is a critical part of metabolism, however, it also produces ROS which leads to damaging cells and contributes to diseases and agieng. The Warburg effect suggests that the levels of different cellular energy metabolism are not the same [28]. Cancer cells mainly produce ATP by a high rate of glycolysis followed by lactic acid fermentation in the cytosol, rather than by a relatively low rate of glycolysis followed by oxidation of pyruvate in mitochondria as in most normal cells [29]. Previous studies have shown that mutations in mtDNA, mitochondrial fusion and fission dysfunction and mitochondria-related active enzyme mutation can cause normal cells transformed into cancer cells [30,31]. The enzymes involved in glycolysis are also the target of many anticancer drugs that inhibit their activities or regulate gene expression [32].

It is well known that ROS produced by aerobic cells during metabolic processes and have important roles in cell signaling and homeostasis [33,34]. However, while undergoing a wide variety of environmental stress, ROS levels can increase dramatically thus leading to oxidative damage to cells [35]. Some studies indicate that ROS levels significantly increased in tumor cells and plays a vital role in the occurrence and development of tumors and also participates in many signaling pathways, such as mitochondrial mediated caspase-dependent apoptotic pathway [36] and regulation of Akt/MAPK-related signaling pathways [37], and even affect the proliferation and differentiation of tumor cells [38–40].

Due to mitochondria being the major sources of ATP or ROS, more and more attention has been focussed on the study of the relationship between energy metabolism and tumor [41–43]. Our present study investigated the mechanism of oxidative stress on the biological energy metabolism of ESCC cells. We used the oxidative stress inducer CoCl_2_ and H_2_O_2_ to treat TE-1 cells to study the effects of different stress on cellular bioenergy metabolism.

First of all, we utilized CoCl_2_ to simulate hypoxic microenvironments. Under hypoxic conditions, HIF-1α is no longer degraded and translocated to the nucleus regulating hundreds of genes, and many of them play important roles in cancer cell metabolism [44]. Our findings demonstrate that CoCl_2_ induced the production of ROS and reduced the expression of mitochondria respiratory chain enzyme complex subunits due to reduced gene transcription and protein synthesis. To further confirm the effects of CoCl_2_ on the energy metabolism of TE-1 cells, we detected the OCR and ECAR to evaluate the ability of TE-1 cells OXPHOS and glycolysis, our results indicate that the mitochondrial respiration was significantly decreased, accompanied by the increase in glycolysis rates. This is consistent with the findings in breast cancer [27]. Additionally, we used the ROS scavenger (NAC) to verify our previous hypothesis, and NAC could dramatically rescue from the effects induced by CoCl_2_, which suggest that tumor cell may undergo the metabolic reprogramming to survive under adverse conditions.

Second, H_2_O_2_ as a widely used inducer of cell damage and apoptosis, is capable of producing large amounts of oxygen free radicals that cause cellular oxidative stress [45,46]. Our data proved that H_2_O_2_ indeed could induce apoptosis and inhibit the expression of mitochondria respiratory chain complex subunits. Moreover, we found that H_2_O_2_ could dramatically suppress the OCR and ATP production and simultaneously inhibited the cellular glycolytic rate. Hence, we proposed that the apoptosis induced by H_2_O_2_ may be due to the energy metabolism dysfunction that is caused by the increase in ROS levels. This was further confirmed that NAC pretreatment significantly abolished H_2_O_2_-induced apoptosis in TE-1 cells. These results demonstrate that oxidative stress caused by H_2_O_2_ leads to severe impairment in TE-1 cell energy metabolism. Moreover, our findings show that H_2_O_2_ mediates apoptosis of TE-1 cells through activating caspase 3 and caspase 9. Therefore, H_2_O_2_ induced the increase in ROS levels, causing cell oxidative damage, mitochondrial dysfunction, and resulting in cell biological energy metabolism disorder, and ultimately induced apoptosis.

In summary, we found that ESCC TE-1 cells exhibit distinct changes in bioenergy metabolism in response to different oxidative stress. Under hypoxic condition, TE-1 cells are able to switch their metabolic phenotype from OXPHOS to glycolysis as an alternative source of biogenetic substrates to maintain cell survival. However, TE-1 cells are unable to reprogram metabolism when they are exposed to H_2_O_2_, and eventually leads to cell death due to energy deficiency. This may provide as a novel strategy for treatment of ESCC, and which is also helpful to solve the problems of drug resistance of ESCC by combination with current treatment and realize the specific targetted therapy of tumor cells.

## Supporting information

**Supplemental Figure 1:** Effects of H_2_O_2_ and CoCl_2_ on TE-1 cell growth. **(A)** TE-1 cells were treated with a gradient concentration of CoCl_2_ (0, 50, 100, 200 μM) for 0, 24, 48, 72 h. **(B)** TE-1 cells were treated with various dose of H_2_O_2_ (0, 100, 200, 400 μM) for 0, 24, 48, 72 h. Cell number was counted by flow cytometry.

**Supplemental Figure 2:** TE-1 cells were pre-treated with a gradient concentration of CoCl_2_ (0, 50, 100, 200 μM) or 200 μM CoCl_2_ and NAC (5, 10 mM) for 24h, and the apoptotic cell rate was determined by using Annexin V FITC/PI cell apoptosis detect kit on the BD Accuri™ C6 Plus System.

## Abbreviations

Cyt. b: Cytochrome b
DCF: Dichlorofluorescein
DCFH-DA: Dichlorodihydrofluorescein diacetate
2-DG: 2-Deoxy-D-glucose
ECAR: extracellular acidification rate
EGFR: epidermal growth factor receptor
ERK: extracellular regulated protein kinases
ESCC: esophageal squamous cell carcinoma
FCCP: carbonylcyanide-p-trifluoromethoxyphenylhydrazone
HIF-1α: hypoxia-inducible factor-1α
HRP: horseradish peroxidase
JNK: c-Jun N-terminal kinase
MAPK: mitogen-activated protein kinase
NAC: N-acetyl cysteine
OCR: oxygen consumption rate
OXPHOS: oxidative phosphorylation
PARP: poly ADP-ribose polymerase
PI: oropidium iodine
PTK: protein tyrosine kinase
qRT-PCR: quantitative real-time PCR
ROS: reactive oxygen species
RPMI--1640: roswell Park Memorial Institute-1640
SOD: superoxide dismutase
TBS-T: TBS-Tween

## References

[1] Jemal A., Bray F., Center M.M., Ferlay J., Ward E. and Forman D. (2011) Global cancer statistics. CA Cancer J. Clin. 61, 69–90 21296855 10.3322/caac.20107

[2] Ferlay J., Shin H.R., Bray F., Forman D., Mathers C. and Parkin D.M. (2010) Estimates of worldwide burden of cancer in 2008: GLOBOCAN 2008. Int. J. Cancer 127, 2893–2917 21351269 10.1002/ijc.25516

[3] Warburg O. (1956) On respiratory impairment in cancer cells. Science 124, 269–270 13351639

[4] Stine Z.E. and Dang C.V. (2013) Stress eating and tuning out: cancer cells re-wire metabolism to counter stress. Crit. Rev. Biochem. Mol. Biol. 48, 609–619 24099138 10.3109/10409238.2013.844093 PMC4063414

[5] Talks K.L., Turley H., Gatter K.C., Maxwell P.H., Pugh C.W., Ratcliffe P.J. (2000) The expression and distribution of the hypoxia-inducible factors HIF-1alpha and HIF-2alpha in normal human tissues, cancers, and tumor-associated macrophages. Am. J. Pathol. 157, 411–421 10934146 10.1016/s0002-9440(10)64554-3 PMC1850121

[6] Semenza G.L. (2003) Targeting HIF-1 for cancer therapy. Nat. Rev. Cancer 3, 721–732 13130303 10.1038/nrc1187

[7] Sumiyoshi Y., Kakeji Y., Egashira A., Mizokami K., Orita H. and Maehara Y, Overexpression of hypoxia-inducible factor-1α and p53 is a marker for an unfavorable prognosis in gastric cancer. Clin. Cancer Res. 12, 5112–5117 16951228 10.1158/1078-0432.CCR-05-2382

[8] Polytarchou C., Hatziapostolou M. and Papadimitriou E. (2005) Hydrogen peroxide stimulates proliferation and migration of human prostate cancer cells through activation of activator protein-1 and up-regulation of the heparin affin regulatory peptide gene. J. Biol. Chem. 28, 40428–40435 10.1074/jbc.M505120200 16199533

[9] López-Lázaro M. (2007) Dual role of hydrogen peroxide in cancer: possible relevance to cancer chemoprevention and therapy. Cancer Lett. 252, 1–8 17150302 10.1016/j.canlet.2006.10.029

[10] Wang J. and Yi J. (2008) Cancer cell killing via ROS: to increase or decrease, that is the question. Cancer Biol. Ther. 7, 1875–1884 18981733 10.4161/cbt.7.12.7067

[11] Rhee S.G. (2006) Cell signaling. H_2_O_2_, a necessary evil for cell signaling. Science 312, 1882–1883 16809515 10.1126/science.1130481

[12] Groeger G., Quiney C. and Cotter T.G. (2009) Hydrogen peroxide as a cell-survival signaling molecule. Antioxid. Redox Signal. 11, 2655–2671 19558209 10.1089/ars.2009.2728

[13] Song J., Li J., Qiao J., Jain S., Mark Evers B. and Chung D.H. (2009) PKD prevents H2O2-induced apoptosis via NF-kappa B and p38 MAPK in RIE-1 cells. Biochem. Biophys. Res. Commun. 378, 610–614 19059215 10.1016/j.bbrc.2008.11.106 PMC2631172

[14] Polytarchou C., Hatziapostolou M. and Papadimitriou E. (2005) Hydrogen peroxide stimulates proliferation and migration of human prostate cancer cells through activation of activator protein-1 and up-regulation of the heparin affin regulatory peptide gene. J. Biol. Chem. 280, 40428–40435 16199533 10.1074/jbc.M505120200

[15] Schieber M. and Chandel N.S. (2014) ROS function in redox signaling and oxidative stress. Curr. Biol. 24, R453–R462 24845678 10.1016/j.cub.2014.03.034 PMC4055301

[16] Veal E.A., Day A.M. and Morgan B.A. (2007) Hydrogen peroxide sensing and signaling. Mol. Cell 26, 1–14 17434122 10.1016/j.molcel.2007.03.016

[17] Florea A.M. and Büsselberg D. (2011) Cisplatin as an anti-tumor drug: cellular mechanisms of activity, drug resistance and induced side effects. Cancers (Basel) 3, 1351–1371 24212665 10.3390/cancers3011351 PMC3756417

[18] Wagner B.A., Evig C.B., Reszka K.J., Buettner G.R. and Burns C.P. (2005) Doxorubicin increases intracellular hydrogen peroxide in PC3 prostate cancer cells. Arch. Biochem. Biophys. 440, 181–190 16054588 10.1016/j.abb.2005.06.015 PMC4538991

[19] Liu Y., Lan L., Huang K., Wang R., Xu C., Shi Y. (2014) Inhibition of Lon blocks cell proliferation, enhances chemosensitivity by promoting apoptosis and decreases cellular bioenergetics of bladder cancer: potential roles of Lon as a prognostic marker and therapeutic target in baldder cancer. Oncotarget 5, 11209–11224 25526030 10.18632/oncotarget.2026 PMC4294382

[20] Shen Y.A., Wang C.Y., Hsieh Y.T., Chen Y.J. and Wei Y.H. (2015) Metabolic reprogramming orchestrates cancer stem cell properties in nasopharyngeal carcinoma. Cell Cycle 14, 86–98 25483072 10.4161/15384101.2014.974419 PMC4352969

[21] Tarrado-Castellarnau M., de Atauri P. and Cascante M. (2016) Oncogenic regulation of tumor metabolic reprogramming. Oncotarget 7, 62726–62753 28040803 10.18632/oncotarget.10911 PMC5308762

[22] Hanahan D. and Weinberg R.A. (2011) Hallmarks of cancer: the next generation. Cell 144, 646–674 21376230 10.1016/j.cell.2011.02.013

[23] Moghadam A.A., Ebrahimie E., Taghavi S.M., Niazi A., Babgohari M.Z., Deihimi T. (2013) How the nucleus and mitochondria communicate in energy production during stress: nuclear MtATP6, an early-stress responsive gene, regulates the mitochondrial FF-ATP synthase complex. Mol. Biotechnol. 54, 756–769 23208548 10.1007/s12033-012-9624-6

[24] Porporato P.E., Payen V.L., Baselet B. and Sonveaux P. (2016) Metabolic changes associated with tumor metastasis, part 2: Mitochondria, lipid and amino acid metabolism. Cell. Mol. Life Sci. 73, 1349–1363 26646069 10.1007/s00018-015-2100-2 PMC11108268

[25] Stefano G.B. and Kream R.M. (2015) Cancer: mitochondrial origins. Med. Sci. Monit. 21, 3736–3739 26621573 10.12659/MSM.895990 PMC4671449

[26] DeBerardinis R.J., Sayed N., Ditsworth D. and Thompson C.B. (2008) Brick by brick: metabolism and tumor cell growth. Curr. Opin. Genet. Dev. 18, 54–61 18387799 10.1016/j.gde.2008.02.003 PMC2476215

[27] Cairns R.A., Harris I.S. and Mak T.W. (2011) Regulation of cancer cell metabolism. Nat. Rev. Cancer 11, 85–95 21258394 10.1038/nrc2981

[28] Liberti M.V. and Locasale J.W. (2016) The Warburg effect: how does it benefit cancer cells ? Trends Biochem. Sci. 41, 211–218 26778478 10.1016/j.tibs.2015.12.001 PMC4783224

[29] Visvader J.E. (2011) Cells of origin in cancer. Nature 469, 314–322 21248838 10.1038/nature09781

[30] Dupuy F., Tabariès S., Andrzejewski S, Dong Z., Blagih J. and Annis M.G. (2015) PDK1-dependent metabolic reprogramming dictates metastatic potential in breast cancer. Cell Metab. 22, 577–589 26365179 10.1016/j.cmet.2015.08.007

[31] Cui X.G., Han Z.T., He S.H., Wu X.D., Chen T.R., Shao C.H. (2017) HIF1/2α mediates hypoxia-induced LDHA expression in human pancreatic cancer cells. Oncotarget 8, 24840–24852 28193910 10.18632/oncotarget.15266 PMC5421893

[32] Pereira W.O. and Amarante-Mendes G.P. (2011) Apoptosis: a programme of cell death or cell disposal? Scand. J. Immunol. 73, 401–407 21223349 10.1111/j.1365-3083.2011.02513.x

[33] Wu X., Deng G., Li M., Li Y., Ma C. and Wang Y. (2015) Wnt/β-Catenin signaling reduces Bacillus Calmette-Guerin-induced macrophage necrosis through a ROS -mediated PARP/AIF- dependent pathway. BMC Immunol. 16, 16 25887795 10.1186/s12865-015-0080-5 PMC4394565

[34] Schieber M. and Chandel N.S. (2014) ROS function in redox signaling and oxidative stress. Curr. Biol. 24, R453–R462 24845678 10.1016/j.cub.2014.03.034 PMC4055301

[35] Cheresh P., Kim S.J., Tulasiram S. and Kamp D.W. (2013) Oxidative stress and pulmonary fibrosis. Biochim. Biophys. Acta 1832, 1028–1040 23219955 10.1016/j.bbadis.2012.11.021 PMC3639303

[36] Sampson V.B., Vetter N.S., Kamara D.F., Collier A.B., Gresh R.C. and Kolb E.A. (2015) Vorinostat enhances cytotoxicity of SN-38 and temozolomide in ewing sarcoma cells and activates STAT3/AKT/MAPK pathways. PLoS ONE 10, e0142704 26571493 10.1371/journal.pone.0142704 PMC4646493

[37] Schumacker P.T. (2006) Reactive oxygen species in cancer cells: live by the sword, die by the sword. Cancer Cell 10, 175–178 16959608 10.1016/j.ccr.2006.08.015

[38] Hamanaka R.B. and Chandel N.S. (2010) Mitochondrial reactive oxygen species regulate cellular signaling and dictate biological outcomes. Trends Biochem. Sci. 35, 505–513 20430626 10.1016/j.tibs.2010.04.002 PMC2933303

[39] Gupta S.C., Hevia D., Patchva S., Park B., Koh W. and Aggarwal B.B. (2012) Upsides and downsides of reactive oxygen species for cancer: the roles of reactive oxygen species in tumorigenesis, prevention, and therapy. Antioxid. Redox Signal. 16, 1295–1322 22117137 10.1089/ars.2011.4414 PMC3324815

[40] Valko M., Rhodes C.J., Moncol J., Izakovic M. and Mazur M. (2006) Free radicals, metals and antioxidants in oxidative stress-induced cancer. Chem. Biol. Interact. 160, 1–40 16430879 10.1016/j.cbi.2005.12.009

[41] Iommarini L., Ghelli A., Gasparre G. and Porcelli A.M. (2017) Mitochondrial metabolism and energy sensing in tumor progression. Biochim. Biophys. Acta 1858, 582–590 28213331 10.1016/j.bbabio.2017.02.006

[42] Troncone M, Cargnelli SM, Villani LA, Isfahanian N., Broadfield L.A. and Zychla L. (2017) Targeting metabolism and AMP-activated kinase with metformin to sensitize non-small cell lung cancer (NSCLC) to cytotoxic therapy; translational biology and rationale for current clinical trials. Oncotarget 10.18632/oncotarget.17496 PMC5593680 28915708

[43] Strickland M. and Stoll E.A. (2017) Metabolic reprogramming in glioma. Front. Cell Dev. Biol. 5, 43 28491867 10.3389/fcell.2017.00043 PMC5405080

[44] Masoud G.N. and Li W. (2015) HIF-1α pathway: role, regulation and intervention for cancer therapy. Acta Pharm. Sin. B 5, 378–389 26579469 10.1016/j.apsb.2015.05.007 PMC4629436

[45] Nogueira V., Park Y., Chen C.C., Xu P.Z., Chen M.L., Tonic I. (2008) Akt determines replicative senescence and oxidative or oncogenic premature senescence and sensitizes cells to oxidative apoptosis. Cancer Cell 14, 458–470 19061837 10.1016/j.ccr.2008.11.003 PMC3038665

[46] Liu J., Chung H.J., Vogt M., Jin Y., Malide D., He L. (2011) JTV1 co-activates FBP to induce USP29 transcription and stabilize p53 in response to oxidative stress. EMBO J. 30, 846–858 21285945 10.1038/emboj.2011.11 PMC3049210

