# Supplementary material for: Oxidative stress regulates cellular bioenergetics in esophageal squamous cell carcinoma cell
Source: Biosci Rep. 2017 Dec 12;37(6):BSR20171006. doi: 10.1042/BSR20171006 (PMC5725616; doi:10.1042/BSR20171006)
Supplement: Supplementary file 1 [file bsr20171006_Supp1.pdf]

Supplemental Figure 1

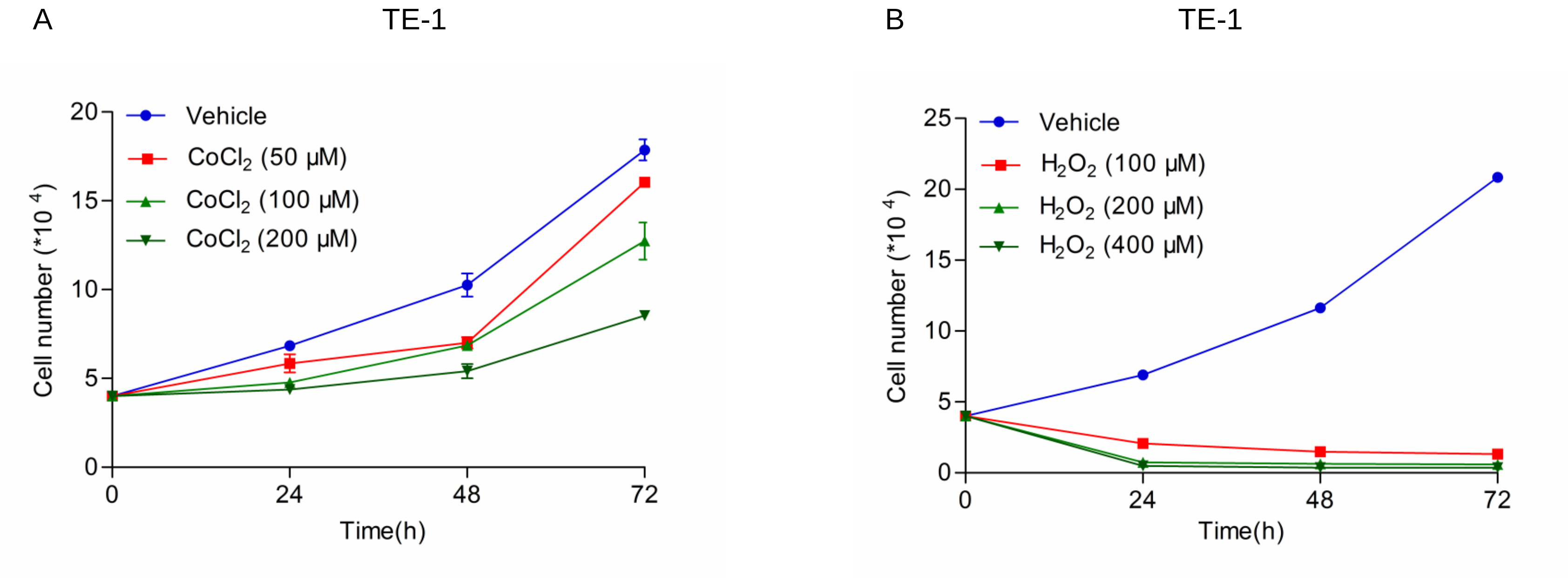

Supplemental Figure 1. Effects of H_2O_2 and $CoCl_2$ on TE-1 cell growth. (A) TE-1 cells were treated with a gradient concentration of $CoCl_2$ (0, 50, 100, 200 μM) for 0, 24, 48, 72 h. **(B)** TE-1 cells were treated with various dose of H_2O_2 (0, 100, 200, 400 μM) for 0, 24, 48, 72 h. Cell number was counted by flow cytometry.

Supplemental Figure 2

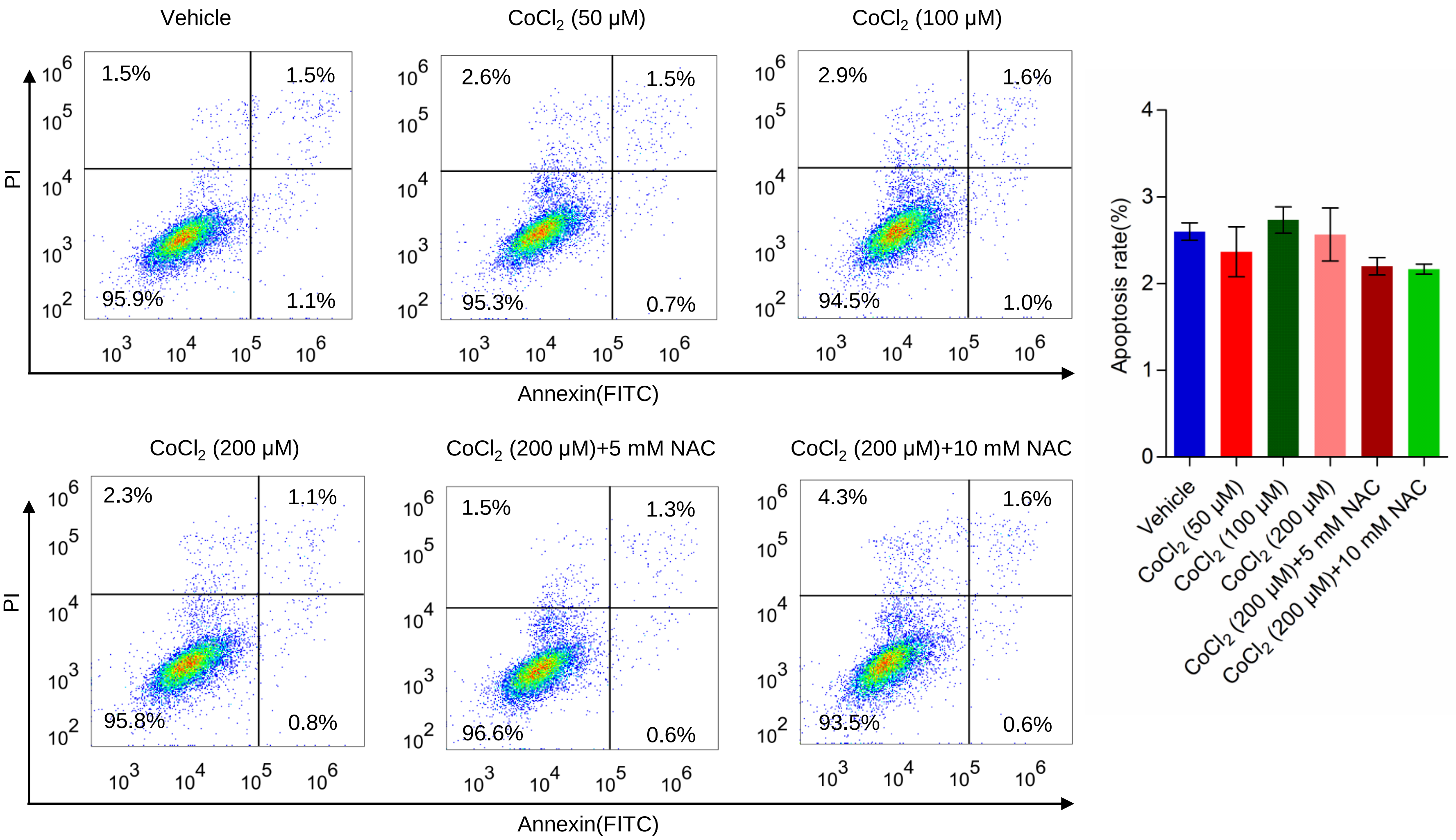

Supplemental Figure 2. TE-1 cells were pre-treated with a gradient concentration of $CoCl_2$ (0, 50, 100, 200 μM) or 200 μM $CoCl_2$ and NAC (5, 10 mM) for 24 h, and the apoptotic cell rate was determined by using Annexin V FITC/PI cell apoptosis detect kit on the BD Accuri™ C6 Plus System.

Supplemental figure 1

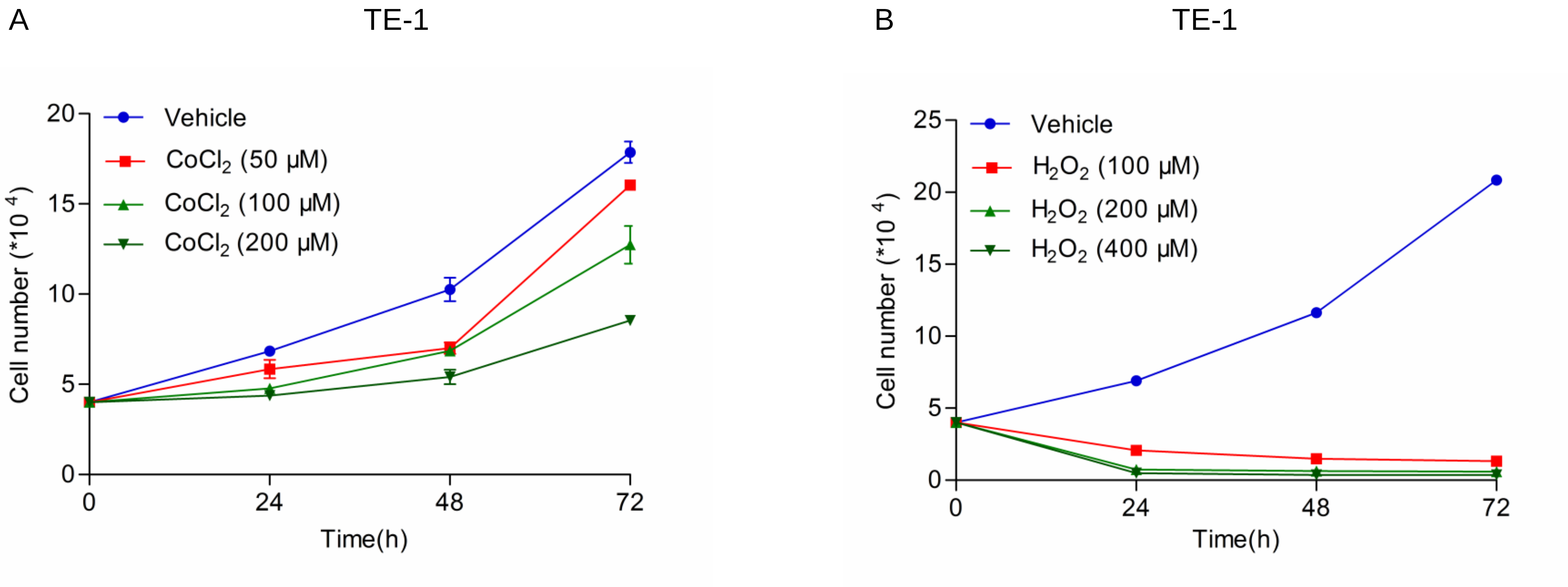

Supplemental Figure 1. Effects of H_2O_2 and $CoCl_2$ on TE-1 cell growth. (A) TE-1 cells were treated with a gradient concentration of $CoCl_2$ (0, 50, 100, 200 μM) for 0, 24, 48, 72 h. **(B)** TE-1 cells were treated with various dose of H_2O_2 (0, 100, 200, 400 μM) for 0, 24, 48, 72 h. Cell number was counted by flow cytometry.

Supplemental Figure 2

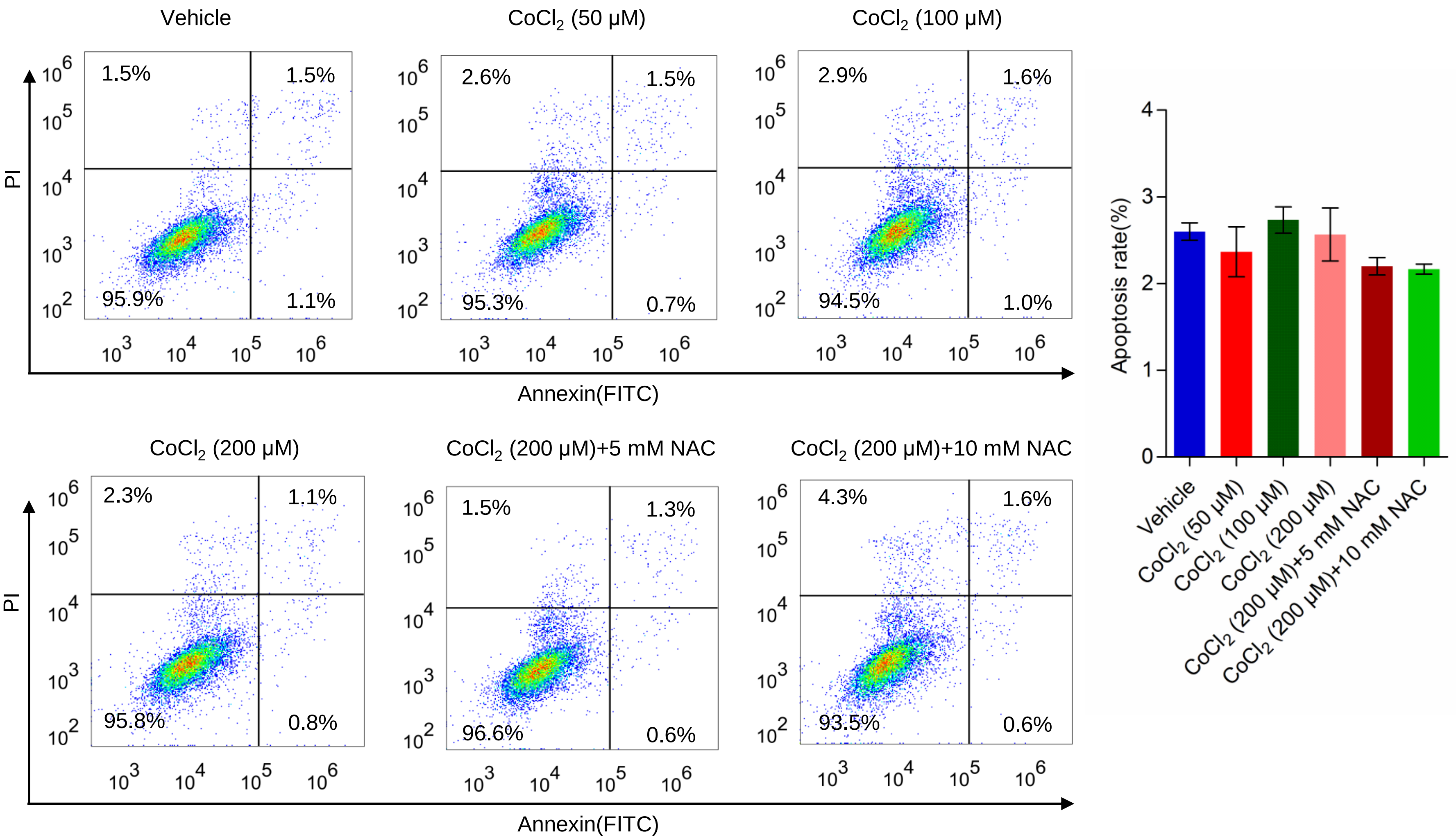

Supplemental figure 2. TE-1 cells were pre-treated with a gradient concentration of $CoCl_2$ (0, 50, 100, 200 μM) or 200 μM $CoCl_2$ and NAC (5, 10 mM) for 24 h, and the apoptotic cell rate was determined by using Annexin V FITC/PI cell apoptosis detect kit on the BD Accuri™ C6 Plus System.
